# Genetic structure provides insights into the geographic origins and temporal change in the invasive charru mussel (Sururu) in the southeastern United States

**DOI:** 10.1371/journal.pone.0180619

**Published:** 2017-07-07

**Authors:** Sávio H. Calazans C, Linda J. Walters, Flavio C. Fernandes, Carlos E. L. Ferreira, Eric A. Hoffman

**Affiliations:** 1 Department de Biologia Marinha, Universidade Federal Fluminense - UFF, Niterói, Rio de Janeiro, Brazil; 2 Department de Oceanography, Instituto de Estudos do Mar Almirante Paulo Moreira - IEAPM, Arraial do Cabo, Rio de Janeiro, Brazil; 3 Department of Biology, University of Central Florida, Orlando, Florida, United States of America; National Cheng Kung University, TAIWAN

## Abstract

In 2004, *Mytella charruana* (d'Orbigny, 1842) (Mollusca: Bivalvia: Mytilidae) became established along the coast of the southeastern United States (SE-US). Using mitochondrial DNA sequencing (cytochrome c oxidase subunit I), we compared genetic variation throughout its native range in South America to its invasive range in the SE-US. Samples from the SE-US were collected in 2006 and 2010 enabling a temporal comparison to evaluate possible genetic changes of the invasive population. We addressed two questions. First, what are the potential source populations (or geographic regions) for the SE-US invasion? Second, how has genetic diversity changed between the two sampling periods within the SE-US? We identified a total of 72 haplotypes, 64 of which were isolated to geographic sites and only 8 were shared among sites. The highly structured native range provides insight into the origin of invasive populations where our results suggest that the introduced SE-US population originated from multiple source populations with the Panama region as the primary source. Additionally, our results indicate that genetic composition of the non-native populations was unchanged between the two sampling periods. *Mytella charruana* exhibit a significant pattern of genetic structure among natural populations, owing to biogeographic barriers that limit natural dispersal, and an ability to persist in novel habitats, owing to a suite of life-history characters that favor survival under variable conditions. Overall, this study explains why *M*. *charruana* may become an increasing threat to locations founded by anthropogenic transportation.

## Introduction

Human activities pose a significant threat to biodiversity and natural ecosystems due to the potential introduction of non-native species [1, 2]. Although the majority of introductions are unsuccessful, when a species becomes established in a novel environment, the consequences can be catastrophic [3] Once a species arrives in a new environment it is likely that other species will follow (i.e. invasional meltdown [4]), further altering the native habitat, either by linked (i.e. pests and diseases [5, 6]) or facilitated association [7, 8]. Given that biological invasions are changing natural systems on a large scale, it is important that we understand general characteristics of species introductions and mechanisms of species invasions [9, 10]. Investigating ecological and genetic questions allow us to illuminate how evolutionary characteristics of invading species favor invasion success and help to understand a species’ potential of invasion [11].

In particular, genetic characterization of invasive populations has proven to be important for several reasons, including species identification [12, 13], distinguishing population of origin [14], and characterizing levels of diversity in the invaded range [15]. A number of studies have shown that non-native populations tend to have greater genetic variation when compared to native populations which may increase the likelihood of establishment [15–17]. Moreover, the high levels of genetic diversity found in invasive populations are usually ascribed to multiple population founders coalescing in the invaded range [15, 17–27].

Despite the detrimental conditions that may follow an invasion, initial colonizers do not always pose a threat when they first settle in an area due to low density and the likelihood that the invasion will fail to flourish [11]. Indeed, during the establishment phase non-native species typically experience a lag-time before they become invasive [28]. During this phase, the introduced population may evolve as an adaptive mechanism to survive in the new environment [29–31]. However, few studies have investigated genetic change over time within the invaded range [29, 32, 33].

Studies seldom have the opportunity to characterize an invasion from its very beginning because of low densities and lack of ecological and economic impact of recent invaders. One exception to this is the invasive charru mussel *Mytella charruana* (d'Orbigny, 1842), a 3-5cm mussel that lives in muddy areas of bays and estuaries forming dense aggregations or covering mangroves roots and other hard substrates [34]. *Mytella charruana* exhibit broad salinity and temperature tolerances, surviving in salinities ranging from 2–40 ppt [35] and temperatures between 6°– 31°C [36].This mussel is native to Central and South America, ranging on the Pacific coast from Guaymas Sonoro, Mexico to Southern Ecuador, and along the Atlantic coast from Colombia to Argentina [37–41]. In 1986, *M*. *charruana* was first discovered in Jacksonville, Florida, USA, attached to a seawater pipe of a power plant [42]. This species was presumably introduced to Florida by ship ballast water or hull fouling from South America [24, 43]. In 1987, after a cold winter, *M*. *charruana* was considered extirpated from the Florida coast. However, in 2004 several individuals were discovered in the Mosquito Lagoon located 212 km south of the original discovery site [44]. Since 2004, *M*. *charruana* has been found along the southeastern coast of the United States from Florida to South Carolina [45] and several studies have investigated ecological aspects of invasion in that area [45–48]. Moreover, a recent study verified negative impacts of *M*. *charruana* on native eastern oysters (*C*. *virginica*), confirming the invasiveness of the species [35, 49].

Molecular genetic studies have found that native populations of *M*. *charruana* along the Brazilian coast were genetically diverse, more structured than other related species (i.e. *M*. *guyanensis* [50]) and resistant to extreme environmental conditions [35, 51]. Samples from the Southeastern United States (i.e. Georgia and Florida–referred to as SE-US from now on) were found to be genetically similar to each other and exhibited higher levels of genetic diversity than and differentiated from native populations [24, 52]. Additionally, studies within the native range have identified an uncommon mitochondrial (mtDNA) evolutionary history of *M*. *charruana*, with three different mtDNA lineages recognized for this species [52]. These three lineages include two maternal lineages and one paternal lineage (i.e. double uniparental inheritance–DUI [53]). Previous studies that have investigated these lineages have been geographically constrained to the east coast of Brazil limiting our understanding of how they exist throughout the species range.

In this study, we analyzed the mtDNA cytochrome c oxidase subunit I (COI) gene of *M*. *charruana* from 12 populations in 7 countries to investigate the relationship between native and nonnative populations of this invasive species. We used these data to address two primary questions: First, what were the possible source populations for the introduced populations? Second, did mtDNA haplotype composition change over time within the invaded region? Considering that a previous study [24] suggested that *M*. *charruana* in the SE-US was of mixed origin with likely sources from the southern Caribbean, we predicted that source populations would originate from the northern region of South America. Moreover, we used these data to investigate why invaded populations do not show marked founder effects (e.g. low genetic variability compared to source population areas) expected with typical new population founding [24, 54]. With regard to our second question, the time-scale investigated here is too short for us to expect to see mutation or selection driven changes in mtDNA markers that might occur over time-periods for quickly evolving loci. Rather, we aimed to see whether new propagules influenced genetic composition owing to the founding of new mussels. We predicted that we would see a change in the genetic composition over time due to fluctuations of population densities in the introduced area. In particular, Spinuzzi et al. [45] found that a sharp die-off of *M*. *charruana* after 2009/2010 extreme cold events throughout the invaded range. Finally, we discuss how spatial and temporal genetic structuring of populations can be used to better understand the initial phases of marine invasions.

## Materials and methods

### Sample collection

**Fig 1:**
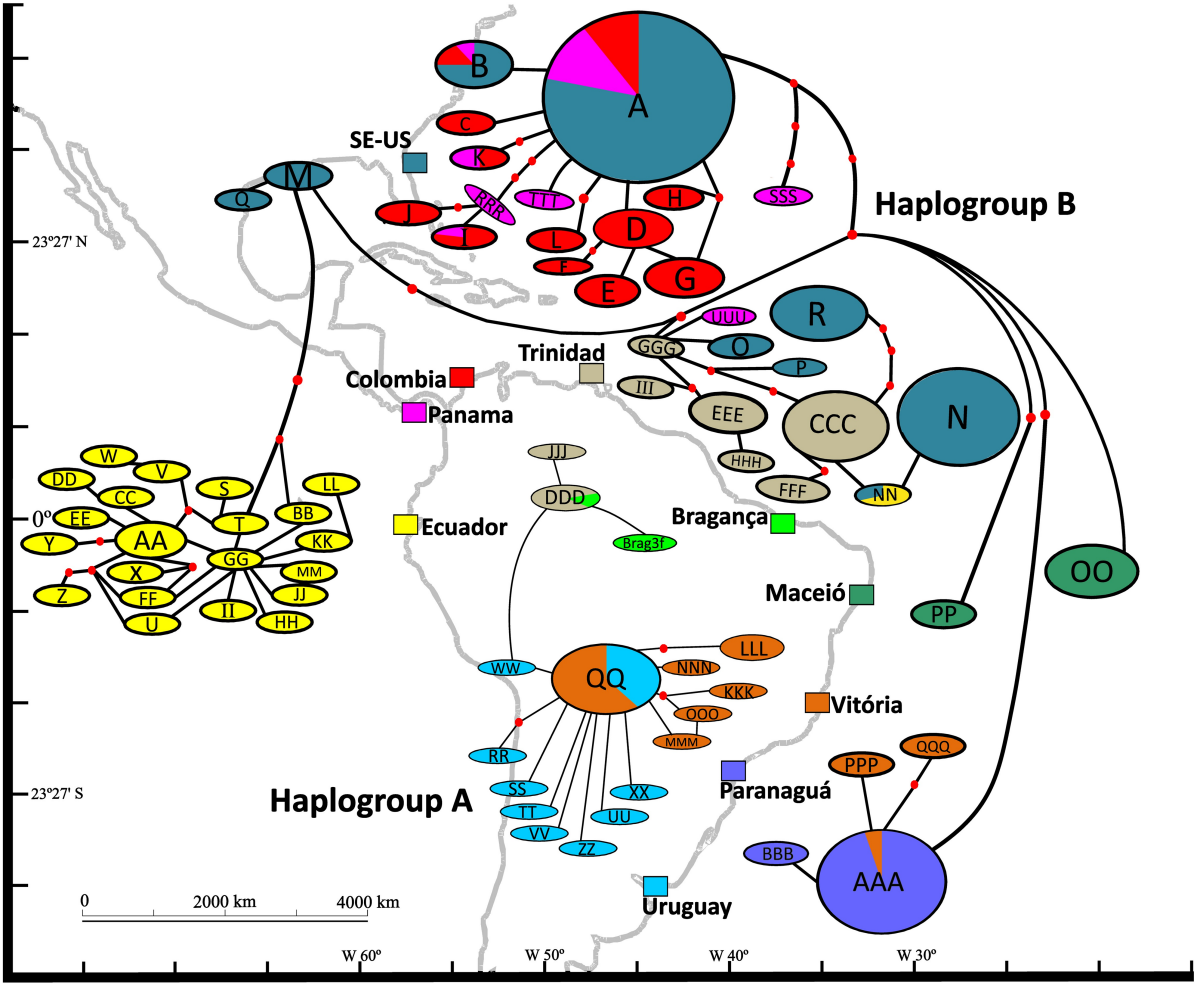
Map of sample sites with overlay of haplotype network of the mtDNA COI gene. Haplotypes were labeled following the naming convention found in Gillis et al. [24]. Circles represent frequency of individuals per haplotype. Colors of site name and haplotype correspond to localities where haplotypes were found. Red nodes represent inferred haplotypes. Note that Lineage A and B haplotypes do not connect into a single network.

For this study, we employed mtDNA haplotypes from six newly sequenced geographic regions from the native range [Panama (PANA), Trinidad & Tobago (TRIN), Northeast Brazil (MACO), Southeast Brazil (VITO), South Brazil (PARA) and Uruguay (MONT) as well as previously published data representing the invaded region (SE-US [24]) with three native regions Ecuador (ECUA), Colombia (COLO [24]) and Northern Brazil (BRAG [53]) to evaluate the genetic relationships within the native region and between native and introduced populations of *M*. *charruana* (Fig 1). The SE-US region [24] is represented by 4 population sample sites: New Smyrna Beach, Florida (NSB), Jacksonville, Florida (JAX), St. Marys, Georgia (SMA), and Sunbury, Georgia (SUN). For all populations, we collected between 30 and 50 samples which were either immediately placed in 100% ethanol or dried in silica gel (i.e. Drierite^™^) for DNA preservation. Additionally, we collected samples from two separate time points for one population (JAX2006 [24] and JAX2010, collected for this study) enabling us to estimate how genetic diversity changed over time. Although recent studies [55, 56] questioned whether *M*. *charruana* was native to Colombia, in this study we consider Colombia to be native given the genetic characterization presented in Gillis et al. [24] and the close proximity to the original species distribution [39]. Sampling in Brazil was carried out under License No. 37119–3 from the Brazilian Chico Mendes Institute for Conservation and Biodiversity. Sampling outside of Brazil was conducted from public docks and required no special permits or permissions for these non-endangered invertebrates.

### DNA extraction and amplification

We amplified a 722 base pair (bp) fragment of the mitochondrial cytochrome c oxidase subunit I (COI) gene using polymerase chain reaction (PCR), with primers originally designed by Gillis et al. [24]. Genomic DNA was extracted from tissue samples of adductor muscle by standard phenol–chloroform methods [57] or using QIAGEN DNeasy Blood & Tissue Kit extraction kits following the manufacturer’s protocol. PCR was performed using the following final concentrations in a 20μL reaction: 2mM MgCl_2_, 0.8mM dNTPs, 1x PCR buffer, 0.5 μM forward and reverse primers (novel primers [24]), 1 unit Taq DNA polymerase, and 50–100 ng DNA template. Cycling conditions were: 95°C for 4 min followed by 35 cycles of 95°C for 30s, 45°C for 30s and 72°C for 30s; final extension period at 72°C for 7 min. PCR products were checked for amplification and size on a 2% agarose gel. All PCR products were purified using exonuclease I and shrimp alkaline phosphatase (ExoSAP; USB, Cleveland, OH, USA) and were submitted for sequencing in both directions to the University of Arizona Genetics Core (Tucson, AZ, USA).

### Data analysis

Sequences were inspected and aligned in Sequencher 4.7 [58]. Open reading frames and changes in amino acids were verified in ExPASy [59] and all sequences were compared to those of Gillis et al. [24] for congruence. Sequences used in this study could not be directly compared to those of Souza et al. [52] given minimal overlap of sequences. However, the overlap that exists enabled us to determine whether our sequences fell into Lineage A or Lineage B of Alves et al. [53] and our sequences are assigned to these groups.

To characterize phylogeographic patterns throughout native and introduced regions, we reconstructed the phylogenetic tree with unique COI haplotypes using the best-fit substitution model and partitioning schemes identified by PartitionFinder v1.1.0 [60]. The phylogenic reconstruction was performed with MrBayes 3.2.6 [61] using two runs of four chains that were sampled every 500 steps for 5,000,000 generations. For each run, the first 2,000 samples were discarded as burn-in. *Mytella guyanensis* was used as an out-group in the phylogenetic reconstruction. Posterior probability values equal to or higher than 95% were considered statistically significant [62]. Because genetic differentiation is not always great enough to supply strong nodal support when investigating intraspecific variation, we employed a statistical parsimony algorithm described by Templeton et al. [63] to construct a 95% haplotype network in TCS v. 1.21 [64].

To investigate the genetic structure of *M*. *charruana* from throughout the native and invasive range, we estimated global and pairwise *F*_ST_ among all populations in Arlequin 3.5.2.2 [65]. Additionally, we estimated gene diversity (*h*) and nucleotide diversity (*π*) in Arlequin and employed a *t*-test in R statistical package version 3.2.3 [66] to evaluate whether genetic diversity within native versus within invasive populations were significantly different. Further, we used molecular analysis of variance (AMOVA) to estimate the differentiation among the four invasive populations and among the eight native populations as well differentiation between natives and invasives. To determine whether native populations exhibited a pattern of isolation-by-distance, we ran a Mantel test with 1000 bootstrap simulations in IBDWS [67]. Geographic distances between populations were estimated with Google Earth 7.1.5.1557 [68] by measuring the least-cost distance along the coastline.

To infer which native population(s) were the most likely source(s) of the introduced SE-US *M*. *charruana*, we used two approaches. First, we determined the number of shared haplotypes between native and invasive populations. Second, we conducted pairwise contrasts between native and invasive populations via analysis of molecular variance (AMOVA). Here, we ran a series of AMOVA analyses in which we grouped the introduced populations with populations from each native region. If these population pairings revealed groupings that were not significantly differentiated between native and introduced populations, then that native population was inferred to be a putative founder population of the invasion. All AMOVA analyses were based on pairwise difference tests with 1000 bootstrap simulations to build confidence intervals for estimations using Arlequin 3.5.2.2 [65].

To address our second question, whether genetic diversity would change over time, we used AMOVA to compare the genetic composition of the 2006 SE-US introduced populations with that of the 2010 JAX population. Here, a significant *F*_*ST*_ value would indicate that genetic variation had changed between the two collection time points. Finally, we estimated gene diversity (*h*) and nucleotide diversity (*π*) in Arlequin for both time points of the invasive region to determine whether diversity indices had changed over time. Here, significance was determined if the 2010 estimates of genetic diversity were outside the 95% CI generated by the four SE-US populations in 2006.

## Results

### Sequence analysis

Our sample set comprised eight populations ranging throughout the native distribution, from Central to South America, as well as five populations from the introduced range along the SE-US (four from 2006 and one from 2010). The average number of sequences per population was 23, with MACO and MONT having the fewest (n = 14) and COLO the greatest (n = 41). A total of 320 sequences of *M*. *charruana* were analyzed using the mitochondrial COI gene. From those, 166 were new sequences and 154 were taken from literature [24]. All sequences were 722 bp long and belonged to the female mtDNA lineage because the novel primers developed by Gillis [24], and used in this study, only amplify COI matrilineal fragments [53]. The number of variable sites within populations ranged from 1 in PARA to 52 in VITO (Table 1). Among the native populations, ECUA and COLO presented the highest genetic diversity (*h* = 0.9) while PARA exhibited the least genetic diversity (*h* = 0.16; Table 1). The highest nucleotide diversity was found in TRIN and VITO (*π* = 0.0189) and was due to the occurrence of haplotypes from both female mtDNA lineages present in these populations (Table 1). Among the invasive populations, all haplotypes belonged to haplogroup B (Table 1). The highest gene diversity (h = 0.825) was found in SMA and the lowest (*h* = 0.702) in JAX2006. The nucleotide diversity ranged from π = 0.00738 (JAX2010) to *π* = 0.00904 (NSB). The entire data set is available in GenBank (Acc. No. MF074963–MF075128).

**Table 1:** Descriptive genetic parameters of *Mytella charruana* populations from western Atlantic and tropical eastern Pacific classified by realm, region and locality. #Haplotypes designates the total number of different haplotypes found in each population. Lineage B designates the number of those haplotypes that belong to lineage B of Alves et al. [53].

Realm	*Subtropical*					*Tropical*						*Subtropical*	*Temperate*
**Region**	Southeast United States					Pacific		South Caribbean		Brazil	Brazil	Brazil	Southern America
										Northeast	Southeast	South	
**Locality**	New Smyrna,* FL^#^ (NSB)	Sunbury GA* (SUN)	St. Marys GA* ^(SMA)^	Jacksonville FL* (JAX2006)	Jacksonville FL* (JAX2010)	Guayaquil Ecuador* (ECUA)	Aguadulce Panama (PANA)	Cartagena Colombia* (COLO)	Trinidad & Tobago (TRIN)	Maceió AL (MACO)	Vitória ES (VITO)	Paranaguá PR (PARA)	Montevideo Uruguay (MONT)
**Latitude**	29°1'32.18"N	31°45'51.77"N	30°42'24.86"N	30°18'52.51"N	30°18'52.51"N	2°42'0.53"S	8°14'35.10"N	10°22'21.58"N	10°39'47.36"N	9°40'23.86"S	20°19'21.40"S	25°30'6.30"S	34°53'17.36"S
**Longitude**	80°55'9.46"W	81°16'41.12"W	81°27'12.44"W	81°37'23.92"W	81°37'23.92"W	80°14'42.48"W	80°29'58.49"W	75°30'33.80"W	1°32'11.08"W	35°46'3.07"W	40°20'21.56"W	48°30'51.68"W	56°12'10.41"W
**Number of sequences**	17	16	16	34	31	30	27	41	35	14	21	24	14
**Variable sites (polymorphic)**	17	17	16	18	12	27	20	18	51	3	52	1	9
**#Haplotypes (Lineage B)**	6 (6)	5 (5)	5 (5)	5 (5)	5 (5)	22 (22)	24 (24)	12 (12)	8 (6)	2 (2)	9 (3)	2 (2)	9 (0)
***h* (st. dev.)**	0.721 (0.087)	0.733 (0.079)	0.825 (0.052)	0.702 (0.052)	0.7462 (0.0434)	0.959 (0.026)	0.880 (0.035)	0.911 (0.020)	0.761 (0.054)	0.440 (0.112)	0.795 (0.077)	0.159 (0.094)	0.835 (0.101)
***π***	0.00904	0.00851	0.00895	0.0087	0.00738	0.00493	0.00631	0.00437	0.01877	0.00183	0.01896	0.00022	0.00178
	**Invasive**					**Native**							

*Data from Gillis *et al*. [24], *h*—Gene diversity, *π*—Nucleotide diversity.

### Phylogenetic relationship and population structure

**Fig 2:**
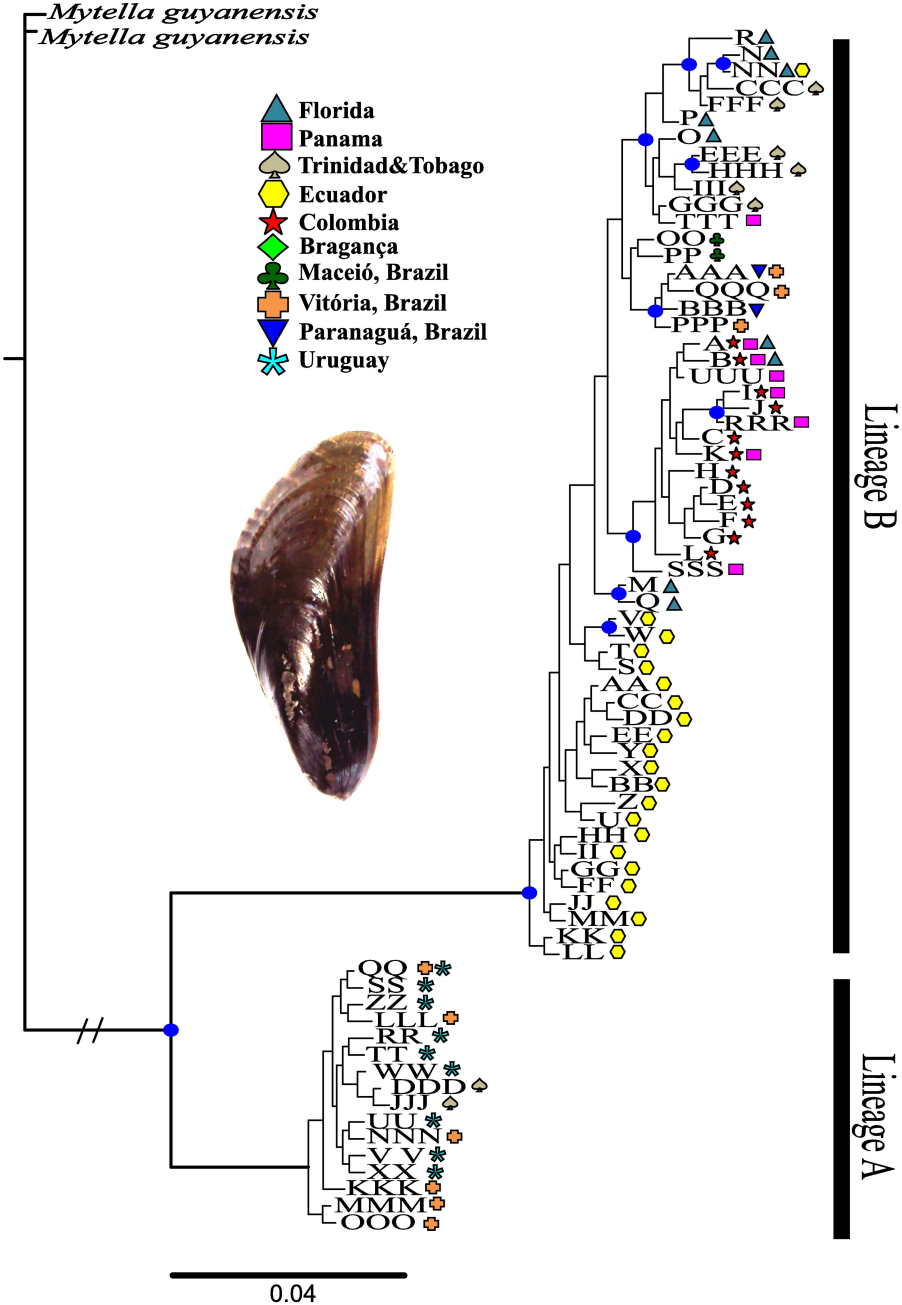
Bayesian phylogenetic tree of Mytella charruana haplotypes using the mtDNA COI gene. The two in-group clades are designated Lineage A and Lineage B [52]. Blue dots designate Bayesian posterior probabilities greater than 0.95. Haplotypes icons are color coded by locality and names correspond to Fig 1.

**Fig 3:**
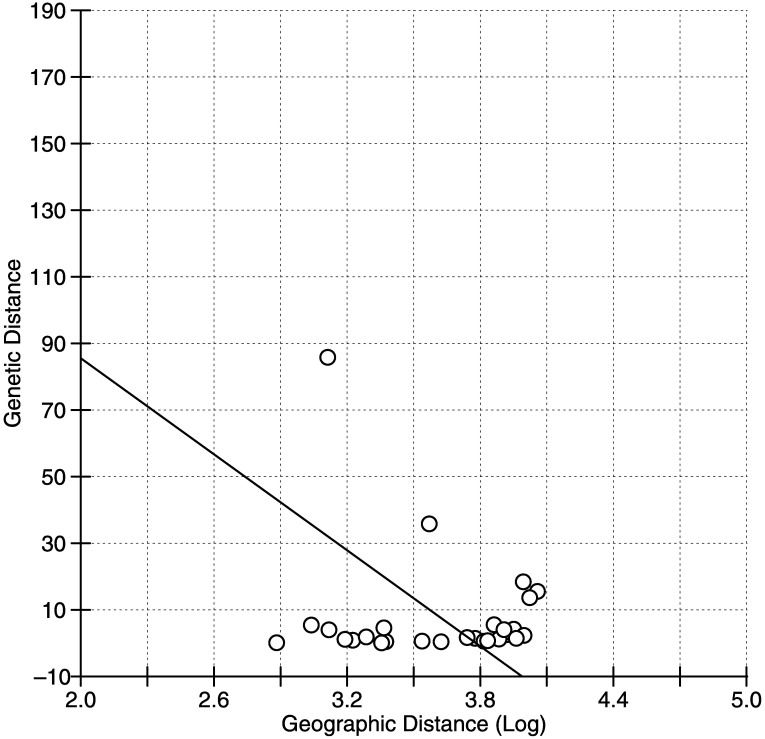
Isolation by distance among native populations. Genetic distance (*F*_ST_/1-*F*_ST_) vs. log of least-cost over-water distance in km (*Z* = 761.9835, *r* = - 0.1576, *p* = 0.765).

The phylogenetic relationship was reconstructed via a Bayesian consensus tree which identified two well defined clades, haplogroup A and B, that had been previously identified by Alves et al. [53] (Fig 2). The best-fit substitution model identified in our analysis was the General Time Reversible model with invariable sites and gamma distribution (GTR + I + G; [69]) and each codon position [K80+I (COI1); F81 (COI2) and HKY (COI3)]. The clade differentiation between groups was well supported by major nodes exhibiting posterior probabilities > 0.95. Similarly, the 95% parsimony network showed haplotype relationships identifying two distinct lineages, with regional genetic differentiation among populations (Fig 1). Lineage B had more haplotypes than Lineage A and contained all haplotypes from the SE-US introduced populations. Additionally, the haplotype network revealed that all haplotypes from ECUA were grouped together, except one (NN) that was grouped with TRIN and SE-US. TRIN and VITO were the only populations that exhibited haplotypes from both Lineage A and B, and MONT was the only population with all haplotypes belonging to Lineage A. TRIN showed a higher number of haplotypes from Lineage B, while VITO had the opposite with more haplotypes from Lineage A. When testing for a relationship between genetic distance and least-cost-coastline distance, we found that native populations did not exhibit a pattern of isolation by distance (Mantel test; *Z* = 761.9835, *p* = 0.765; *r* = - 0.1576; Fig 3).

**Fig 4:**
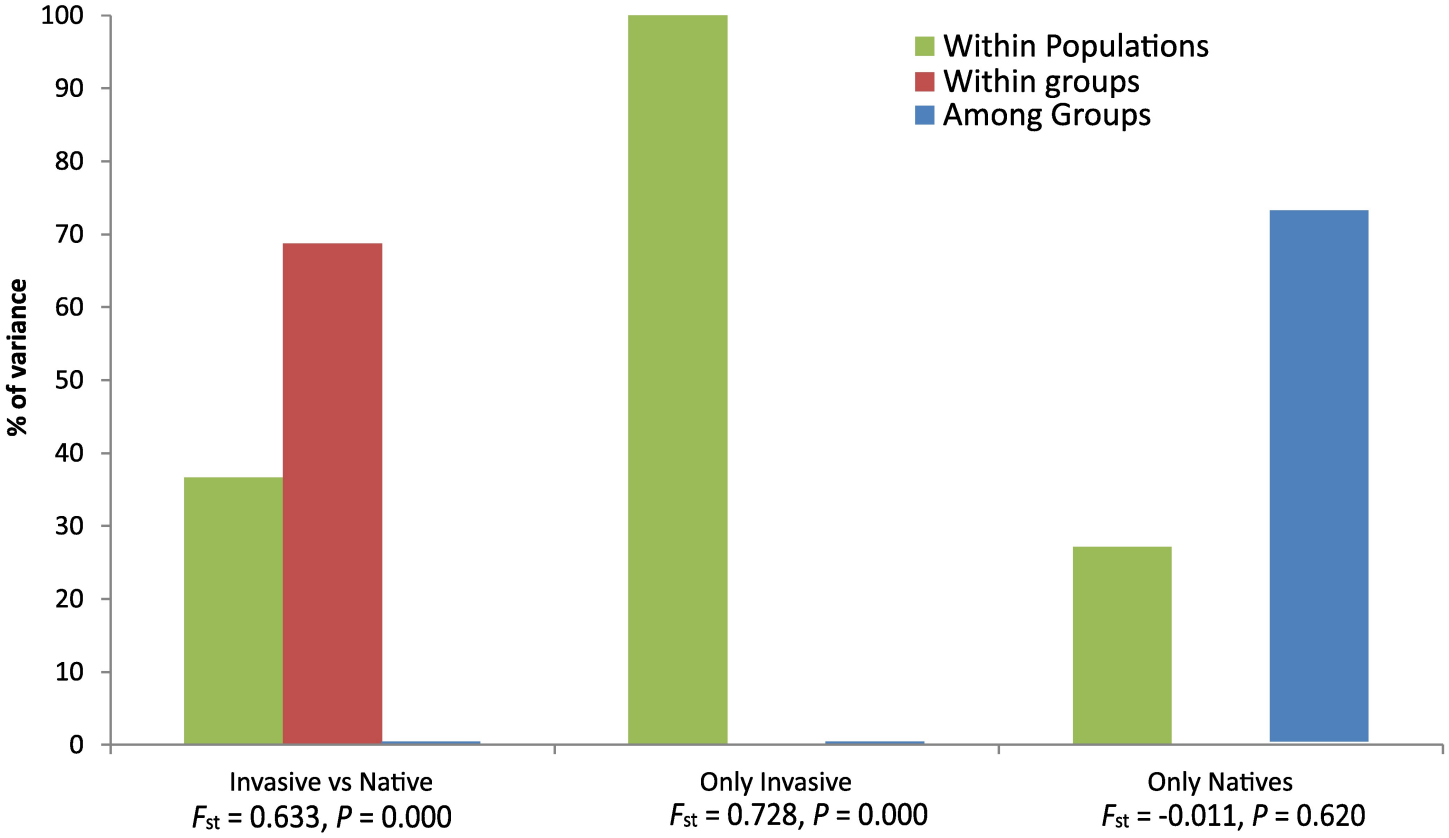
Graphs of the results from three analyses of molecular variance (AMOVA). Each analysis is separated by populations included in the analysis (invasive versus native populations, invasive populations only, and native populations only). For each analysis, columns indicate the percent of variance explained. Below columns are global *F*_ST_ and *p*-values for a null hypothesis of no genetic structure.

Genetic differentiation between the native and the invasive region showed significant structuring (*F*_ST_ = 0.633; Fig 4). Specifically, comparisons between all native populations were marked by high *F*_ST_ values (global *F*_ST_ = 0.728), and a large component of variation among localities (Fig 4 –Only Natives). Of 61 haplotypes found in native populations, only seven were shared among any eight native population (A, B, I, K, between PANA and COLO; AAA, between VITO and PARA; QQ between VITO and MONT and DDD between TRIN and BRAG) and the remainder were private haplotypes and typically closely related to other haplotypes from the same regions (Figs 1 and 2). In pairwise analyses, the lowest estimate of *F*_ST_ between native populations was between MONT and VITO (*F*_ST_ = 0.074), whereas the highest estimate of *F*_ST_ was between PARA and MONT (*F*_ST_ = 0.988). In contrast, introduced populations were not significantly differentiated from each other (global *F*_ST_ = -0.011, *p* = 0.602, Fig 4). Between introduced populations, pairwise *F*_ST_ estimates were predominantly negative with the highest value between JAX2010 and NSB (*F*_ST_ = 0.049). Values contrasting genetic diversity between native and invasive populations were not significantly different for either gene diversity (*p* = 0.791) or nucleotide diversity (*p* = 0.6187) (Table 1).

### Source of invasion and temporal changes

**Fig 5:**
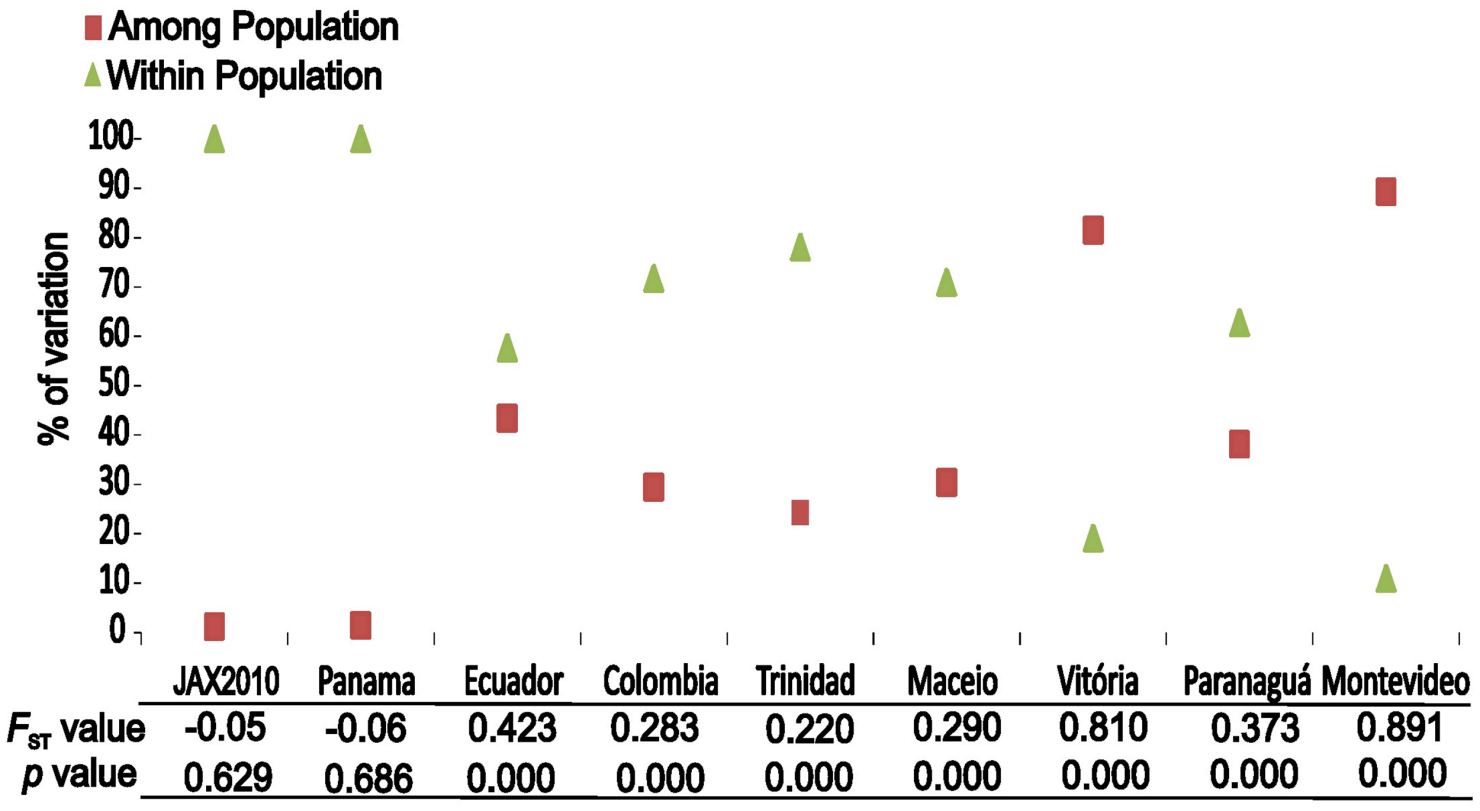
Plots of multiple analyses of molecular variance (AMOVA) grouping Southeast-US 2006 with each native population and with the JAX2010 population. Below each plot are global *F*_ST_ and the *p*-values testing the null hypothesis of no genetic structure.

Given that native populations exhibited genetic differentiation from each other and that our collected samples spanned throughout the native distribution of *M*. *charruana*, we ran independent AMOVA analyses to determine the degree of differentiation between native and non-native populations, as a means to identify if any native population could be a potential founder of the SE-US populations. We found that only a single native population exhibited a non-significant *F*_ST_ (PANA, *F*_ST_ = -0.06, *P* = 0.686; Fig 5) when contrasted with SE-US populations. All other native populations exhibited significant genetic differentiation relative to the SE-US populations (Fig 5). The percent of variance explained among regions in the AMOVA comparisons between native populations and the SE-US populations was smallest for PANA (as expected given the non-significant *F*_ST_ between these populations), then followed by the northern South American populations (TRIN, COLO, MACO; Fig 5). Other native populations exhibited significant variance explained by regions comparing the native versus non-native regions (Fig 5). Our direct comparisons of haplotype composition were largely congruent with the AMOVA comparisons. We observed nine distinct haplotypes in the SE-US populations (A, B, M, N, O, P, Q, R, NN) of which three (A, B, NN) were shared with native populations (PANA, ECUA and COLO).

To determine whether there was a change in the genetic composition of populations over time in the invaded region, we ran an AMOVA contrasting the SE-US populations from 62006 with the JAX2010 population. This analysis revealed that the estimate of *F*_ST_ between these populations was low and not significant (*F*_ST_ = -0.05, *p*-value = 0.629), showing a lack of change over the four-year period (Fig 5). A comparison of haplotypes between sampling periods identified only a single haplotype (NN) which appeared in the JAX2010 sampling period, but was absent in SE-US populations from 2006. Moreover, there were four haplotypes present in 2006 that were absent in 2010 (M, P, Q, R; Figs 1 and 2). Similar to the genetic differentiation data, genetic diversity estimates exhibited little differentiation between sampling periods. Gene diversity for JAX2010 was 0.7462 and fell within the 95% CI of gene diversity from the 2006 SE-US populations (95% CI of *h* = 0.747–0.744), nucleotide diversity for JAX2010 was 0.0074 and fell slightly below the confidence interval found in 2006 SE-US populations (95% CI of *π* = 0.00881–0.00879).

## Discussion

Our genetic assessment revealed a strong patterns of genetic structure throughout native *M*. *charruana* populations. In contrast, variation among invasive populations along the southeastern coast of the United States exhibited little differentiation, even when contrasted with temporally displaced samples on either side of a major population die-off. Diversity within native populations exhibited an order of magnitude in variation among populations, but this level of variation was driven by populations either fixed for a single haplotype group or populations that exhibited mingling of haplotype groups. The genetic diversity of introduced SE-US population remained high considering all invasive populations were composed of a single haplotype group. Overall, compiling genetic information from throughout the native range provided the necessary data to determine that the introduced populations are likely closely related to Pacific Panamanian populations. Likewise, occurrence of shared haplotypes between northern South America and the SE-US provides further evidence that the introduced range included samples of mixed origin. Furthermore, admixture of haplotypes from Panama and southern Caribbean populations explains why within-population diversity is so high for the non-native populations.

Marine organisms have the potential to disperse throughout large areas where genetic isolation is generated with increasing distance [70, 71] and dispersal over large distances can occur with sufficient time and stochastic events [72]. However, determining rates of connectivity and genetic structuring in the marine environment has proven to be challenging and more complex than previously assumed. Although genetic structuring should reflect major biogeographic barriers (e.g. Isthmus of Panama Barrier (IPB); Amazon Barrier (AB); Mid-Atlantic Barrier (MAB); and the Benguela Barrier (BB) [73]), barriers for dispersal do not affect all species equally [72]. There are examples of benthic species with long-range dispersal [73–77] and mobile species that exhibit fine-scale genetic structuring [78, 79]. Likewise, long-term pelagic larval duration does not always cause species to exhibit a lack of structure over a large geographic range [80]. Indeed, dispersal by marine organisms depends not just on pelagic larval duration but also on habitat requirements [81] and evolutionary history of a species [82]. Although the length of pelagic larval duration is not directly related to genetic structuring for benthic invertebrates, long-range dispersal frequently occurs during the pelagic larval phase in benthic taxa [83, 84, 85].

Connectivity among populations of estuarine taxa would be expected to be reduced compared to marine and coastal taxa [86, 87]. In estuarine environments, structuring is dependent on the process of migration from one estuary to another. Given that estuaries are often separated by open ocean segments, estuarine environments tend to restrict gene flow, driving populations to local adaptation and boosting genetic differentiation among estuarine taxa [86]. Examples of estuarine taxa that exhibit significant genetic structuring include: amphipod (*Gammarus zaddachi* [88]); gastropod mollusk (*Hydrobia ventrosa* [89]); catfish (*Cnidoglanis macrocephalus* [90]); echinoid (*Evechinus chloroticus* [91]); shrimp (*Macrobrachium nipponense* [92]); as well the sister taxa to *M*. *charruana*, *M*. *guyanensis* [50]. *M*. *charruana*, as a typical estuarine mussel, exhibits a high degree of isolation throughout the native distribution, as demonstrated here.

In addition to the estuarine environment leading to differentiation, ocean currents are another feature along the South American coast that also likely contributes to differentiation among populations [93, 94]. Ocean currents and associated differences in water temperature are known to impact genetic structure throughout the Atlantic (e.g. king weakfish, *Macrodon ancylodon*, exhibit deep genetic divergence correlated with the northern flow of the Brazilian Current and the Malvinas Current, despite morphometric homogeneity [94]) and other oceans (e.g. *Perna perna*, [95]). With regard to *M*. *charruana*, we can see the impacts of currents driving differentiation between the populations of PANA and ECUA where the intervening habitat is ideal for *M*. *charruana* (e.g. mangroves and mud flats [96]). These populations are likely genetically differentiated owing to the flow of the Panama Current moving in a southward direction and the Humboldt (Peruvian) Current moving Antarctic water northward. At the confluence, located between PANA and ECUA, the coastal water forms a constant vortex and the current moves westward into the open ocean [97]. Similar to *M*. *charruana*, black mangroves (*Avicenia germinans*) exhibit high population genetic structuring between populations in the northern and southern Pacific coasts of Colombia owing to these currents [98].

Given these barriers to natural dispersal found for *M*. *charruana* throughout the native range, what processes likely brought *M*. *charruana* to the SE-US? Anthropogenic translocation by ship transportation (ballast water and/or hull biofouling) from South America appears to be the most plausible vector for *M*. *charruana*. Many examples from other organisms support ballast water and ship hull fouling as efficient dispersal vectors for marine organisms [99–101], especially considering the intense shipping traffic around Florida waters [102] and previous investigations of the *M*. *charruana* SE-US invasion [24]. Given the characterization of genetic structure from throughout the native distribution conducted by this study, we were able to identify the likely source of the SE-US populations as being from, or near, the Pacific coast of Panama. Certainly new occurrence data from additional Caribbean populations could provide additional insight into the pathway(s) used by *M*. *charruana* to arrive in Florida from Panama.

Within the invasive range, there are both abiotic and biotic factors that can impact the spread of *M*. *charruana*. A recent study investigating temperature and salinity tolerances of *M*. *charruana* sheds light on how abiotic factors limit spread throughout the invaded range [103]. These researchers found that *M*. *charruana* survival was largely driven by temperature given that survivorship curves dropped off quickly at both low and high temperature extremes. With regard to biotic factors, *M*. *charruana* was found established on intertidal oyster reefs in the Indian River Lagoon, Florida [24]. Given that in the SE-US, *M*. *charruana* has only been found on hard substrates, further spreading may be directly related to oyster density. However, a recent study investigating competitive interactions between *M*. *charruana* and oysters (*Crassostrea virginica*) found that oyster spat survival and growth was reduced by the presence of *M*. *charruana* [104]. Native predators have also been found to limit invasive species distribution. For example, Dudas et al. [105] found that native crabs preferentially preyed upon non-native clams over native clams in choice experiments. Unpublished data has found that blue crabs (*Callinectes sapidus*) native to the SE-US equally prey upon *M*. *charruana* and the native ribbed mussel (*Geukensia demissa*) (L. Walters, unpublished data). Overall, there is a paucity of studies evaluating how biotic, abiotic, and the combination of these factors may impact the future spread of *M*. *charruana* throughout the SE-US. However, as climate change impacts North America, the range of invasive *M*. *charruana*, currently believed to be held in check by cold winter temperatures [45], will likely be able to expand into more northern latitudes.

Furthermore, by comparing genetic diversity and differentiation over a temporal scale that spanned the 2009/2010 [45] cold weather event, where the air temperature in Jacksonville, FL fell to 0°C or below for 12 of 13 days (2 January–14 January, 2010 [106]), we were able to address the ephemeral nature of the SE-US population. Our prediction was that the high summer occurrence data found by Spinuzzi et al. [45] could be caused by recurrent re-introductions leading to genetic change over time. Previous investigations of genetic change over time in other species have found mixed results, with some studies reporting genetic diversity to be static [29, 33] while others found that population genetic makeup does change over time [11, 29, 33, 107]. However, our results were counter to our predictions in that we identified a persistent set of haplotypes belonging to the SE-US invasive population of *M*. *charruana*. These results indicate that, even during periods when *M*. *charruana* were not observed, some individuals were protected in stronghold locations and persisted to re-establish the coast when favorable conditions returned.

In sum, this study illuminates how an understanding of genetic differentiation from throughout the native range can provide insights into the spread and persistence of an invasive species. With regard to *M*. *charruana*, the differentiation identified among populations in the native range provided the necessary resolution to identify where the recent southeastern United States invasion originated. Moreover, this study identified an interesting contrast in dispersal patterns for *M*. *charruana*. Specifically, this species exhibits a pattern of minimal dispersal throughout the native range, yet is both persistent (this study) and spreading [45] throughout the invasive range. The life history characters that favor survival in the novel range (e.g. high salinity tolerance [35, 107, 108], high temperature tolerances [35, 36], and the ability to change sex under stressful conditions [46]) cannot overcome the natural geographic barriers found throughout the native range. Synthesizing studies characterizing *M*. *charruana* in both the native and invasive range, we suggest that although *M*. *charruana* will not likely be transported long distances on its own, anthropogenic transportation of this species can lead to foundation events in a variety of habitats that will likely be difficult to eradicate.
