# Correction: Genetic structure provides insights into the geographic origins and temporal change in the invasive charru mussel (Sururu) in the southeastern United States

**DOI:** 10.1371/journal.pone.0195159

**Published:** 2018-03-26

**Authors:** Sávio H. Calazans C, Linda J. Walters, Flavio C. Fernandes, Carlos E. L. Ferreira, Eric A. Hoffman

There is an error in Fig 4. Please see the corrected Fig 4 here.

**Fig 4:**
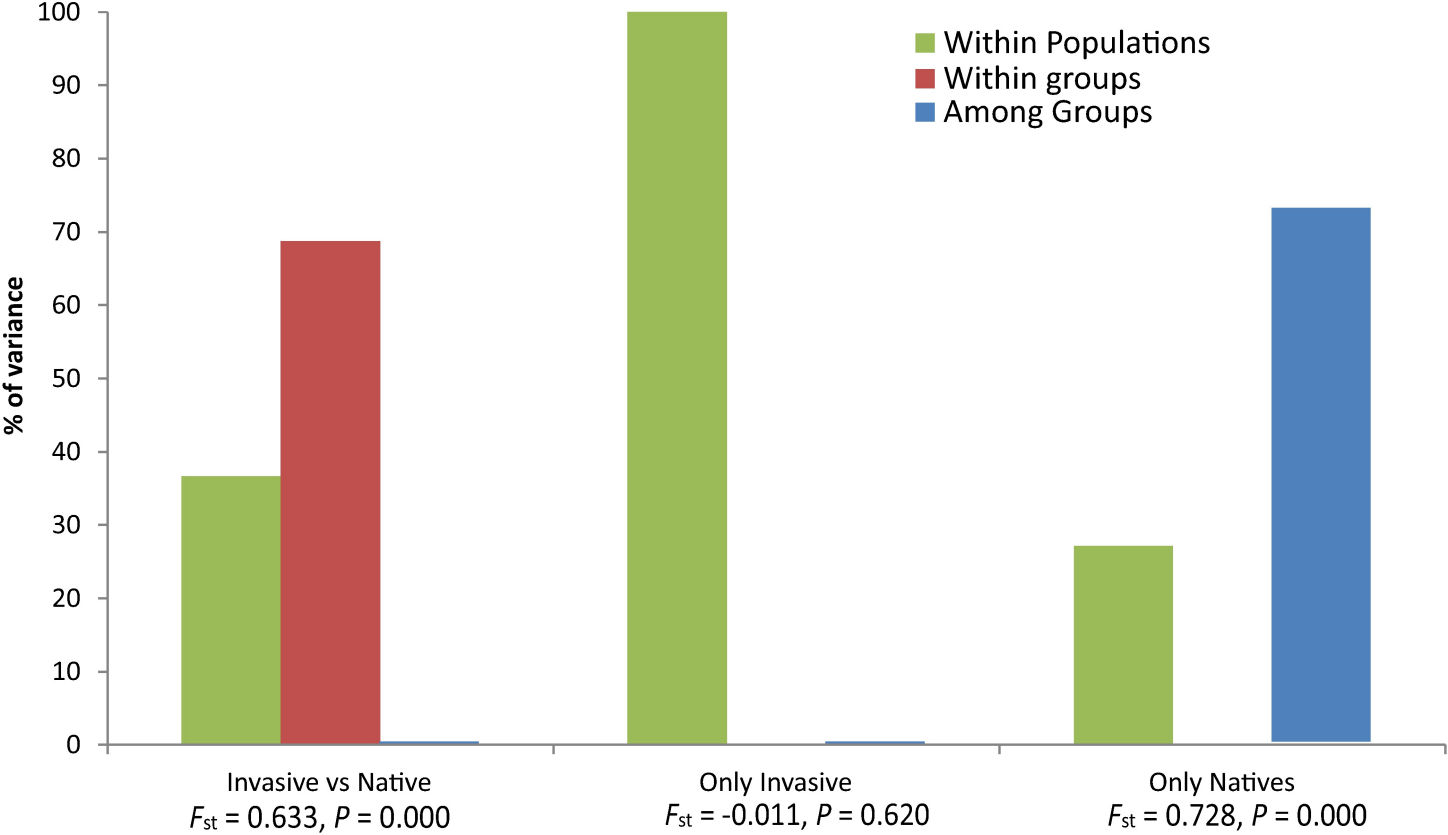
Graphs of the results from three analyses of molecular variance (AMOVA). Each analysis is separated by populations included in the analysis (invasive versus native populations, invasive populations only, and native populations only). For each analysis, columns indicate the percent of variance explained. Below columns are global *F*_ST_ and *p*-values for a null hypothesis of no genetic structure.
